# Non-Invasive Assessment of PVA-Borax Hydrogel Effectiveness in Removing Metal Corrosion Products on Stones by Portable NMR

**DOI:** 10.3390/gels7040265

**Published:** 2021-12-14

**Authors:** Valeria Stagno, Alessandro Ciccola, Roberta Curini, Paolo Postorino, Gabriele Favero, Silvia Capuani

**Affiliations:** 1 Earth Sciences Department, Sapienza University of Rome, Piazzale Aldo Moro 5, 00185 Rome, Italy; 2 Physics Department, National Research Council—Institute for Complex Systems (CNR-ISC), Sapienza University of Rome, Piazzale Aldo Moro 5, 00185 Rome, Italy; silvia.capuani@isc.cnr.it; 3 Department of Chemistry, Sapienza University of Rome, Piazzale Aldo Moro 5, 00185 Rome, Italy; alessandro.ciccola@uniroma1.it (A.C.); roberta.curini@uniroma1.it (R.C.); 4 Department of Physics, Sapienza University of Rome, Piazzale Aldo Moro 5, 00185 Rome, Italy; paolo.postorino@roma1.infn.it; 5 Department of Environmental Biology, Sapienza University of Rome, Piazzale Aldo Moro 5, 00185 Rome, Italy

**Keywords:** portable NMR, PVA-PEO-borax hydrogel, porous stones, coin corrosion products, raman spectroscopy, SEM-EDS

## Abstract

The cleaning of buildings, statues, and artworks composed of stone materials from metal corrosion is an important topic in the cultural heritage field. In this work the cleaning effectiveness of a PVA-PEO-borax hydrogel in removing metal corrosion products from different porosity stones has been assessed by using a multidisciplinary and non-destructive approach based on relaxation times measurement by single-sided portable Nuclear Magnetic Resonance (NMR), Scanning Electron Microscopy—Energy Dispersive Spectroscopy (SEM-EDS), and Raman Spectroscopy. To this end, samples of two lithotypes, Travertine and Carrara marble, have been soiled by triggering acidic corrosion of some copper coins in contact with the stone surface. Then, a PVA-PEO-borax hydrogel was used to clean the stone surface. NMR data were collected in untreated, soiled with corrosion products, and hydrogel-cleaned samples. Raman spectroscopy was performed on PVA-PEO-borax hydrogel before and after cleaning of metal corrosion. Furthermore, the characterization of the dirty gel was obtained by SEM-EDS. The combination of NMR, SEM-EDS and Raman results suggests that the mechanism behind the hydrogel cleaning action is to trap heavy metal corrosion products, such as Cu^2+^ between adjacent boron ions cross-linked with PVA. Moreover, the PVA-PEO-borax hydrogel cleaning effectiveness depends on the stone porosity, being better in Carrara marble compared to Travertine.

## 1. Introduction

The surface cleaning of works of art composed of stone materials is a critical concern for conservators and restorers. The cleaning process involves the removal of dirt, dust, pollutants, metal ions, or microorganisms. In particular, all these agents continuously endanger the life of stone artworks exposed to outdoor. When a stone building, statue or monument under atmospheric conditions is in contact with metallic parts, such as clamps, pivots, or plaques [1], corrosion of the latter may be induced and the corrosion products may hinder the correct readability of the artwork [2,3,4]. In particular, in the case of copper and its alloys, corrosion is a chemical attack mainly promoted by the affinity of metals and pollutants (i.e., sulfur, carbon dioxide, chlorides). This process leads to a corrosive layer called patina [5,6], which can be protective (noble patina) or unprotective (vile patina) [7,8,9], depending on the concentration of pollutants and acid rain. As a result, geographic location, precipitation, and pollution level all have an impact on patina composition and morphology [9,10]. So, the atmospheric exposure of copper produces the oxidation-reduction reactions leading to different corrosion products: copper(I) oxide which is red, copper(II) oxide which is black, black copper sulfide, various colored salts and nantokite, green-blue atacamite and clinoatacamite [6]. These corrosion products are responsible for the discoloration of the stone [1,11].

In the case of iron in contact with a stone artwork and in the presence of oxygen and water, it will corrode depending on the pH value of the surrounding environment [1,4,11]. Generally, corrosion is activated by acidic conditions, but it can also take place in an alkaline environment [11]. The result of iron corrosion will not be a patina adhered to the metal surface but a powdery rust layer produced by electrochemical processes [6]. So, the rust consists of the stratification of the oxides, usually green hydrated magnetite, black anhydrous magnetite and, only externally, the ferric hydroxides [6], which are responsible for cervices formation in the stone surrounding the metallic part [11]. Moreover, also when iron is covered by lead a damage to the stone should be expected because lead itself can be attacked [11]. Behind the surface alterations of stone artefacts induced by the corrosion of metallic components, other factors can cause the aesthetical modification of the artwork. Among these factors, there are old restorations and protecting interventions, deposition of particulate and pollutants, graffiti, and vandalism [12]. Specifically, because of the well-known interaction among SO_2_, PM_10_ and rain pH, black crusts are the most common alteration for stone artworks in cities [9,13]. The black crusts composition reflects that one of the air in which the artwork is exposed and they lead to a mechanical, aesthetical and chemical damage [9].

In this scenario, the cleaning process and the choice of the cleaning substance are of fundamental importance. In the last years, gels or gel-like systems have been widely employed for the cleaning procedure of stone artworks and cultural heritage in general [1,14,15,16,17]. Gels or gel-like systems, as well as high-viscous-polymeric-dispersion [18], have shown great potential due to their high selectivity, low toxicity, and low environmental impact [16,18,19]. Among these, there are gel-systems based on polyvinyl alcohol (PVA). PVA is a water soluble and biocompatible polymer with good resistance to mechanical stress, capable to form hydrogen bonds, thanks to its hydroxyl groups, and ion complexes [19,20,21]. It has emerged as a potential adsorbent because of its high swelling capacity and its resistance to dissolution, mainly due to the formation of cross-links between network chains [22]. In particular, it can capture contaminants entrapping them between the fine pores of hydrogel developed via crosslinking networks [23]. The high-water-content and porous structure networks help to diffuse the solute with contaminants [24]. Moreover, it has been used together with both natural and synthetic compounds to produce different types of hydrogels, which may also be physically or chemically cross-linked. One of these, the PVA-borax hydrogel, obtained by cross-linking of PVA with borate ions [18,20,21,25], can be described as a viscoelastic dispersion with a dynamic network. In fact, the increase in borax concentration expands the system network due to electrostatic repulsions in the polymer chain [20,25,26]. When PVA-borax aqueous gel is applied on the surface of cultural heritage for cleaning purposes, this should be able to remove degradation products by capillarity absorption through their pores [19]. To this end, Riedo et al. [20] studied the effect of polyethylene oxide (PEO) addition to the PVA-borax hydrogel. The authors showed that PEO, which is a water-soluble and biocompatible thermoplastic polymer, would increase the pore size of the system in agreement with other studies [27,28]. Moreover, PEO seemed to improve the mechanical properties of the gel and the retention of its liquid phase [20,29].

PVA-PEO-borax gels, as well as all the gel-systems, can be easily removed in one step from a surface simply by peeling. This represents a great advantage for the conservation treatment of cultural heritage. However, the ease of gel removal, together with its capability of retention of the liquid phase and the absence of gel residues also depend on the characteristics of the surface to be cleaned [12,29].

In this work the cleaning effectiveness of a PVA-PEO-borax hydrogel in removing metal corrosion products from two different porosity stones has been assessed by using a multidisciplinary and non-destructive approach, combining the single-sided portable Nuclear Magnetic Resonance (NMR) to investigate stone samples cleaning, with Scanning Electron Microscopy—Energy Dispersive Spectroscopy (SEM-EDS) to characterize the gel composition after the stone cleaning process and Raman Spectroscopy measurements to study the gel before and after the cleaning procedure.

To this end, samples of two lithotypes, Travertine and Carrara marble, have been soiled by triggering the acidic corrosion of some copper coins in contact with the stone surface. Then, a PVA-PEO-borax hydrogel was used in order to clean the stone surface. NMR relaxation times were evaluated in untreated and treated samples. Pure gel and dirty gel obtained after sample cleaning were analyzed by Raman Spectroscopy. Moreover, the composition of the dirty gel was obtained by SEM-EDS. The novelty of this study is the use of portable NMR as a non-invasive and non-destructive tool for the monitoring of the gel cleaning procedure. Indeed, the NMR protocol that we developed can be employed for in situ analyses to evaluate the cleaning efficacy and action of different gels on different materials.

## 2. Results and Discussion

### 2.1. NMR Characterization of Travertine and Carrara marble

**Figure 1:**
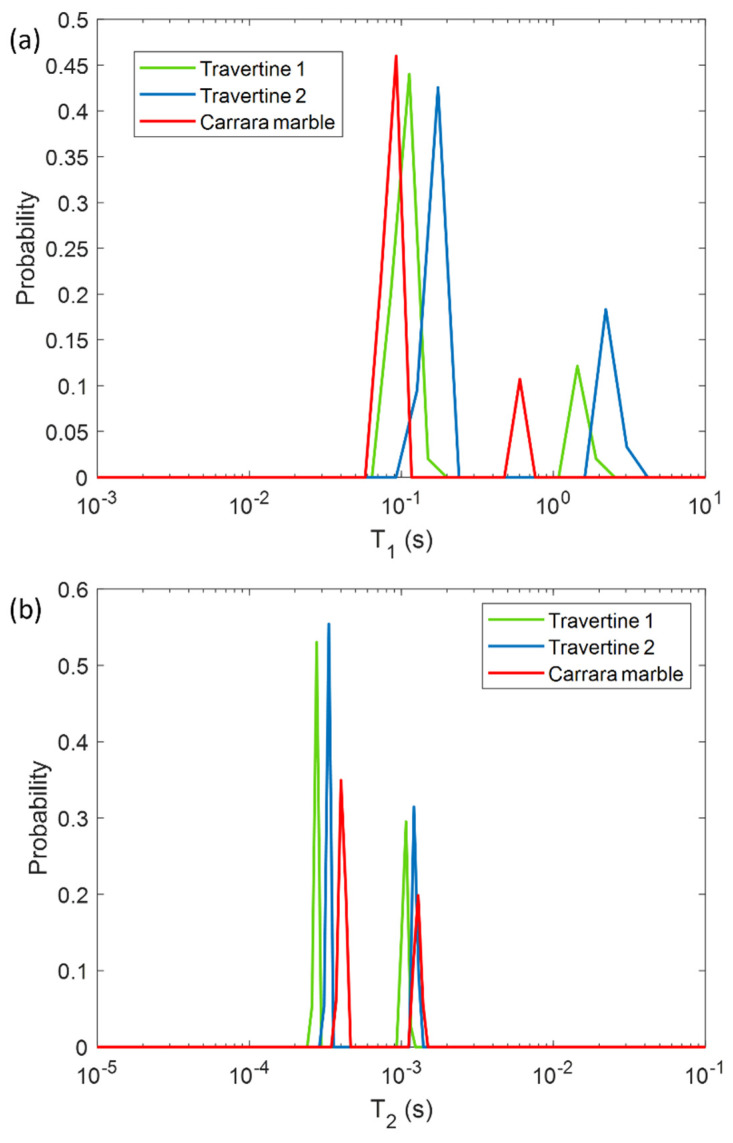
Longitudinal relaxation time *T*_1_ (**a**) and transversal relaxation time *T*_2_ (**b**) distribution for the three untreated samples analyzed in this study: two Travertines (green and blue lines), and one Carrara marble (red line). Travertine 1 refers to the untreated sample before being soiled with corrosion products from penny, whereas Travertine 2 and Carrara marble refer to the untreated samples before being soiled with corrosion products from euro cents.

In a first phase, we characterized the untreated samples of Travertine and Carrara marble with the aim of differentiating the samples on the base of their porosity and morphology, by using NMR *T*_1_ and *T*_2_ parameters. In Figure 1 the *T*_1_ and *T*_2_ relaxation time distributions obtained for the three untreated samples are shown. All the samples are characterized by two *T*_1_ and two *T*_2_ components associated with two different pore size compartments. Regarding the *T*_1_ relaxation time, Travertine 2 is characterized by the higher values (blue curve in Figure 1a), whereas Carrara marble the smaller ones (red curve in Figure 1a). On the other hand, Carrara marble is characterized by the highest *T*_2_ values (red curve in Figure 1b).

In Figure 1, the different *T*_1_ and *T*_2_ mean values among the two Travertine samples and Carrara marble, can be explained on the basis of their different porous structure. Due to the inverse relationship between *T*_2_ or *T*_1_ and the surface-to-volume ratio (S/V) of the pores in a porous medium [30], our results can provide information about the different porosities of the three analyzed samples. The two *T*_1_ and *T*_2_ components detected for all three samples, suggest that two main different pore size compartments exist in the stones. In contrast to our previous work [12], we investigated samples at relative humidity (RH) value equal to 50 ± 3%. For this reason we did not detect the long *T*_2_ component (around few or tens of ms) obtained in the study performed at RH = 94% [12]. Indeed, at RH = 50% less water molecules in vapor form wet the stone pores compared to those present at RH = 94%. About the two Travertine samples, they were cut from the same slab and parallel to the bedding planes. Despite having the same chemical composition, the two Travertines show a rather different pore structure inside the 2 mm layer studied by portable NMR. Indeed, Travertine 2 shows higher *T*_1_ and *T*_2_ than Travertine 1. This result indicates that Travertine 2 pores are characterized by a smaller S/V than those of Travertine 1. Concerning the Carrara marble sample, its *T*_1_ is the smallest, whereas its *T*_2_ is the highest (see red-line in Figure 1a,b). In the Carrara marble, a very few structure metal ions are present, which are responsible for the characteristic color of its veins [31]. Therefore, the shorter *T*_1_ values of Carrara marble displayed in Figure 1a may be attributed to the effect of paramagnetic ions on *T*_1_ [32,33,34].

On the other hand, the longest *T*_2_ values of Carrara marble inform about its pores, which have the smallest S/V. These results suggest that Travertine has larger pores than Carrara marble, in agreement with the literature [31,35].

### 2.2. NMR Monitoring of the PVA-Gel Cleaning of Metal Corrosion Products from Stones Surface

**Figure 2:**
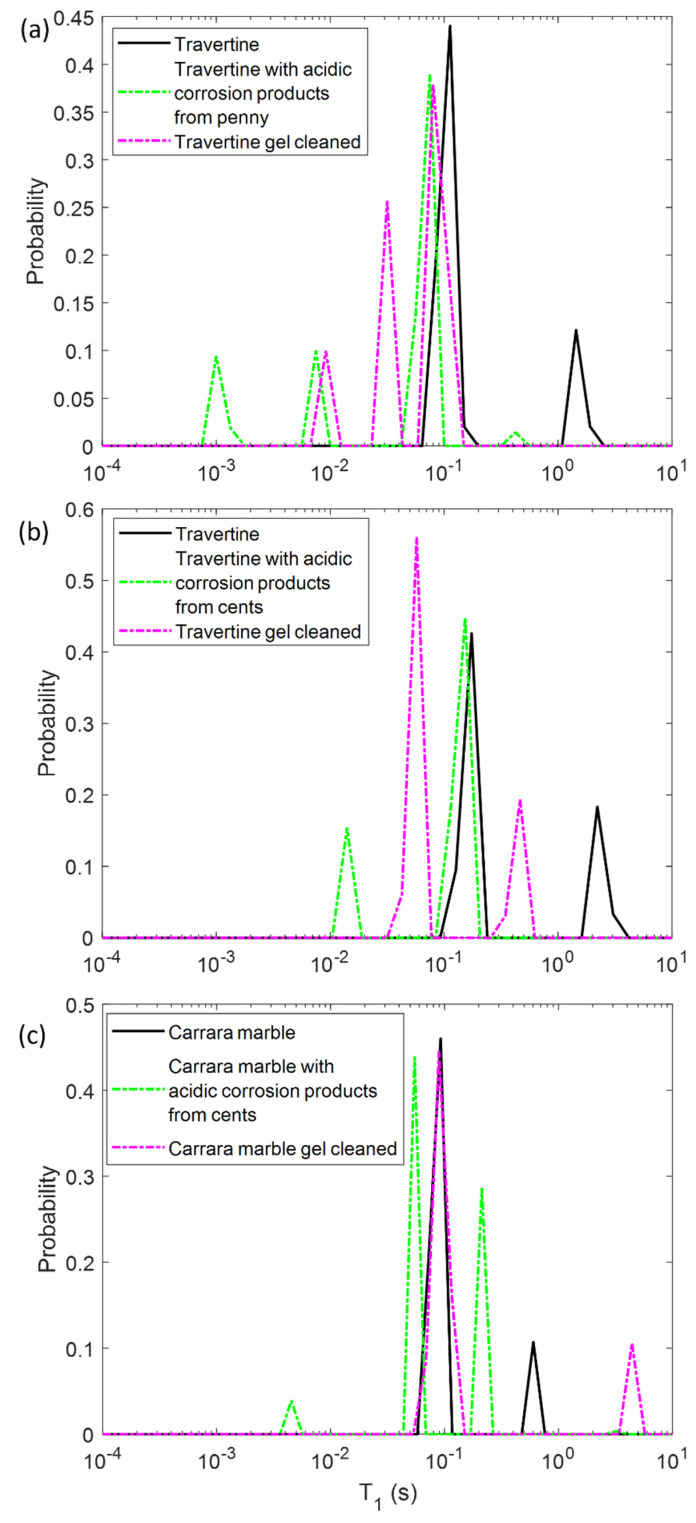
Longitudinal relaxation time *T*_1_ distribution for (**a**) Travertine 1, (**b**) Travertine 2 and (**c**) Carrara marble before (solid-line) and after the soiling process (green dashed-line), and after the gel cleaning (pink dashed-line).

**Figure 3:**
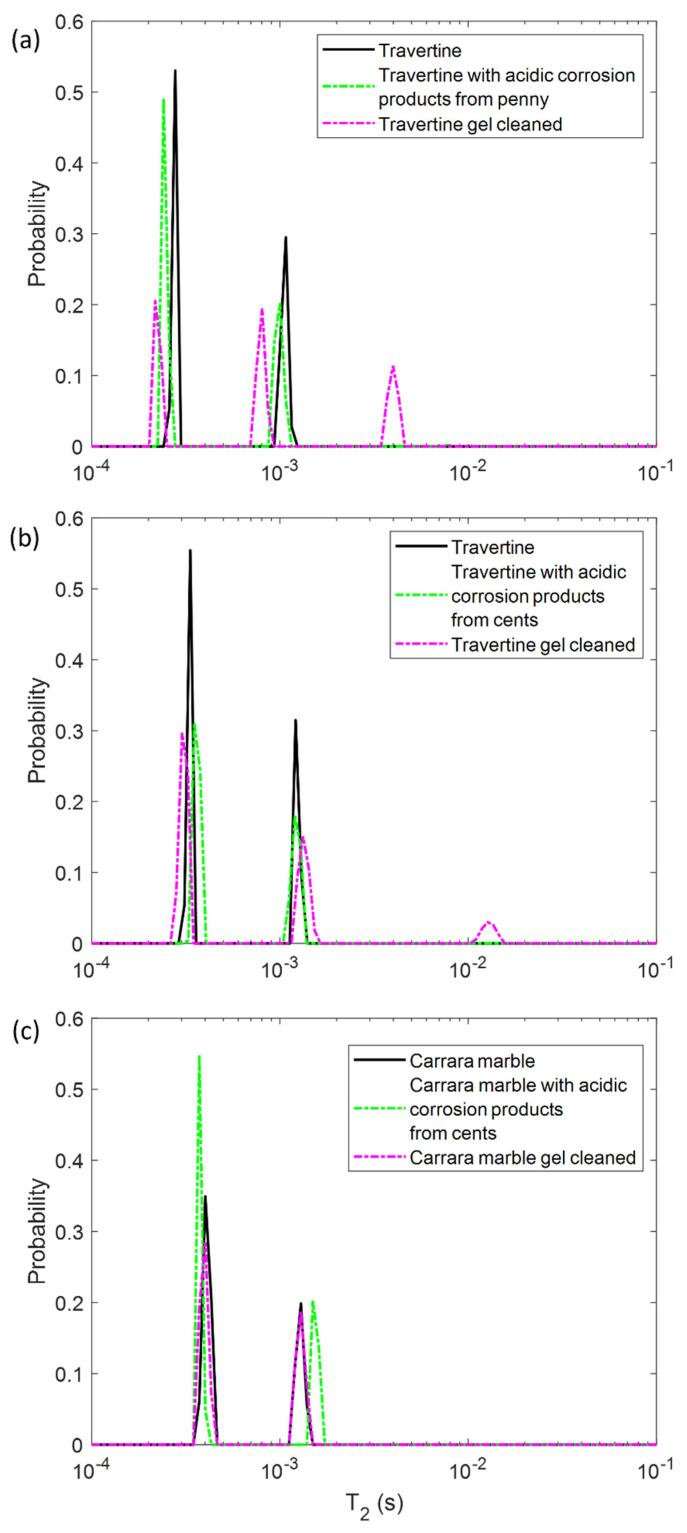
Transversal relaxation time *T*_2_ distribution for (**a**) Travertine 1, (**b**) Travertine 2 and (**c**) Carrara marble before (solid-line) and after the soiling process (green dashed-line), and after the gel cleaning (pink dashed-line).

In the first step, we performed NMR measurements on the soiled samples in order to test if the *T*_1_ and *T*_2_ parameters were affected by the presence of metal corrosion products and if they could inform us about different degrees of soiling of the samples. Then, we repeated the same experiments on the stone surface after the hydrogel cleaning process, with the aim of highlighting the cleaning efficacy and detecting possible gel residues. To this end, in Figure 2 and Figure 3 the *T*_1_ and *T*_2_ distributions before (black solid-line) and after the soiling process (green dashed-line), and after the cleaning process (pink dashed-line) for the two Travertines (Figure 2a,b and Figure 3a,b) and the Carrara marble sample (Figure 2c and Figure 3c) are displayed. Figure 2 and Figure 3 show how the presence of metal corrosion products on the stones surface affects the NMR relaxation times (green-dashed lines). The longitudinal relaxation time *T*_1_ seems to be strongly influenced by the heavy metal corrosion products and it significantly decreases in all the three stained samples compared to the untreated ones (see Figure 2). This result is in agreement with the literature [32,33,34] about the shortening effect of paramagnetic ions on the *T*_1_. Conversely, *T*_2_ is less affected by metal ions. The two main *T*_2_ components of Travertine soiled with penny corrosion products are reduced compared to the untreated sample (see Figure 3a) confirming the already observed effect of paramagnetic ions. Travertine soiled with corrosion products from euro cents in Figure 3b, is characterized by *T*_2_ that is not significantly affected by the presence of metal corrosion products. Furthermore, Carrara marble (Figure 3c) shows one short *T*_2_ component, which is shortened because of the metal corrosion products, and one long *T*_2_, which seems to increase. These different results can be explained on the basis of the different degree of corrosion of the coins used. Indeed, Travertine soiled with corrosion products from the penny has the highest variation in its relaxation times because of the greater degradation state of the already aged penny, which led to a greater deposition of metallic corrosion products (see Section 4.1).

In principle, PVA-gel cleaning should result in a return of the NMR relaxation times to their values measured on the untreated samples. After the gel cleaning, the *T*_1_ of the two Travertine surfaces are slightly increased compared to those ones measured before cleaning. Nevertheless, their *T*_1_ did not return to its original value, acquired on the untreated surfaces. This result may suggest that the PVA-based hydrogel did not completely remove the metal corrosion products from the stone. In addition, the fact that the *T*_1_ did not return to the initial values may also suggest that the gel used has left residues in the pores, as observed in previous studies [12,29]. In particular, in Travertine soiled with corrosion products from the penny (Travertine 1) the *T*_1_ and *T*_2_ distributions have the same behavior with both *T*_1_ and *T*_2_ mean values that did not return to their original value after the cleaning process (Figure 3). While the *T*_1_ components (Figure 2a) measured after the gel cleaning increased compared to those of the soiled surface, the *T*_2_ components did not change (Figure 3a). Moreover, a third component of *T*_2_ around 4 ms was detected after the gel cleaning, probably due to the gel residues (observable by the naked eye, see Section 4.1) or to the removal of dust from the stone surface. A similar consideration can be formulated for the second sample of Travertine (Travertine 2) soiled with corrosion products from euro cents (Figure 2b and Figure 3b). Here, after the gel cleaning, both the *T*_2_ components are close to the initial values measured on the untreated surface (black-solid line). Again, the third *T*_2_ component around 13 ms may suggest gel residues or dust removal. However, because of the very small variation of the *T*_2_ distribution in Travertine 2, our discussions must be considered as preliminary considerations, and further measurements will be performed in future work.

In Carrara marble, after the gel cleaning, both the *T*_1_ and *T*_2_ returned close to their original values measured on the untreated surface (see Figure 2c and Figure 3c). This result indicates that the PVA-borax gel cleaned the Carrara marble surface without leaving residues. However, in Figure 2c the second *T*_1_ component is higher than that one of the untreated sample. This effect can be ascribable to a deep dust removal probably present in the untreated samples. The absence of gel residues in Carrara marble can be explained by the lack of macropores, inside which the gel can penetrate and becomes difficult to remove by peeling, compared to Travertine.

### 2.3. Raman Spectroscopy of the Gel Layers Removed after Cleaning of the Stones Surface

The purpose of Raman analyses was to detect structural variations of the dirty gel (i.e., used for the stone cleaning from metal corrosion products) compared to the pure gel, likely induced by the formation of new bonds among metal corrosion products and the hydrogel polymers.

**Figure 4:**
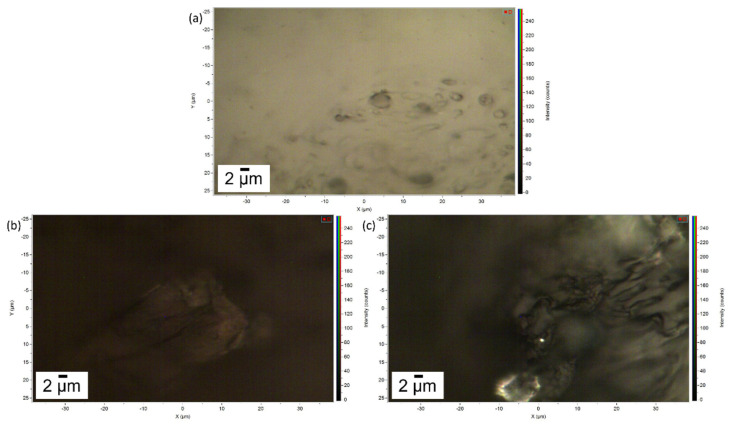
Image of (**a**) the pure gel, (**b**) the gel used to remove penny corrosion products from Travertine and (**c**) the gel used to clean Carrara marble from euro cents corrosion products. The scale bar is 2 µm and the image magnification is 100×.

In Figure 4 the images of the pure gel and of the two layers of gel removed after cleaning of the Travertine and Carrara marble surface are displayed. Figure 4a shows the porous structure of the gel and a brighter appearance compared to the gel used to clean Travertine from penny corrosion (Figure 4b) and Carrara marble from euro cent corrosion (Figure 4c). This result suggests that the PVA-PEO-borax hydrogel interacted with the metal corrosion products.

**Figure 5:**
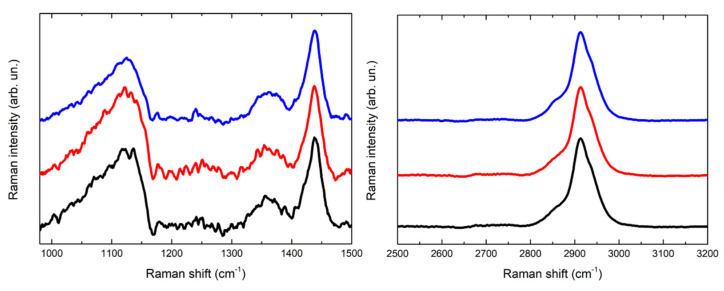
Comparison of three Raman spectra acquired for the reference of PVA-borax hydrogel.

**Figure 6:**
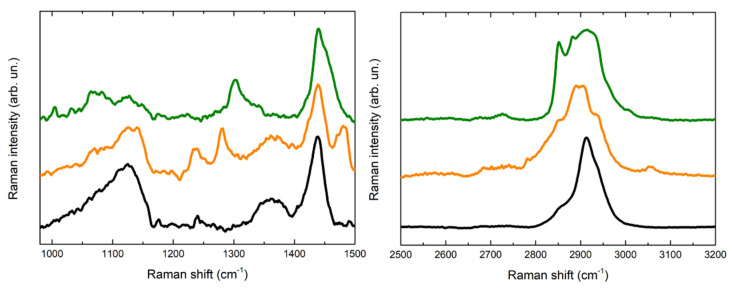
Comparison of Raman spectra acquired for the reference of PVA-borax hydrogel (black line), the cleaning gel for the cent stains on Carrara marble (orange line) and the cleaning gel for the penny stains on Travertine (green line).

In Figure 5 and Figure 6 the Raman spectra acquired are displayed. The spectra acquired for the PVA-borax hydrogel show a great reproducibility (Figure 5), where all the features of the chemical matrices are observable and summed up in Table 1: the broad signal at 1125 cm^−1^ is attributable to B-O-C bond groups, while the intense signal at 1440 cm^−1^, along with the lower intensity band at 1355 cm^−1^, is related to the C-H bending modes in the PVA moieties. At higher wavenumbers, it is possible to observe a main signal at 2913 cm^−1^, characteristic of C-H stretching along with the shoulders at around 2855 and 2935 cm^−1^. These spectral features confirm the evidences reported in literature about PVA and PVA-borax hydrogels [36,37,38,39]. Regarding the hydrogel used for the cleaning of metal corrosion products (Figure 6), some aspects must be highlighted.

First, it is relevant to emphasize that no particles of corrosion products are visible on the hydrogel surface, while the whole matrix is homogeneously colored (Figure 4). For this reason, the spectra were acquired at different random points on the surface. Some differences in the spectra are observable in comparison to the PVA-borax reference spectra. In particular, in one of the spectra acquired from the hydrogel used for the cleaning of the penny corrosion, the band at 1125 cm^−1^ is split into two signals at 1072 and 1127 cm^−1^, while the band at 1355 cm^−1^ disappears and it is replaced by another signal at 1304 cm^−1^. A new band at around 1656 cm^−1^ appears, while, in the higher wavenumber range, defined peaks at 2852, 2882 and 3007 cm^−1^ overlap with the C-H stretching of the original hydrogel matrix. At two points, instead, the main signals of the gel are present, but two peaks at 972 and 1273 cm^−1^ also appear.

Analogously, in the Raman spectrum of the hydrogel used for the cleaning of the euro cent stains on Carrara marble, new signals at 1239, 1282 and 1481 cm^−1^ are visible, while in the C-H stretching range the signal broadens at lower wavenumbers, with the appearance of a shoulder at around 2854 cm^−1^ and a low intensity peak at 3055 cm^−1^. Moreover, even if it is less evident than for the previous cleaning gel, a shoulder at around 1070 cm^−1^ is visible close to the B-O-C signal at 1125 cm^−1^.

Considering the reproducibility of Raman spectra of the hydrogel matrix reference and the absence of visible particles attributable to corrosion products, it is possible to hypothesize that the spectral variations observable in the case of the hydrogel samples used for the cleaning could be attributed to the removal process, which should not have a mechanical origin but probably chemical, otherwise the corrosion products would be clearly visible as aggregates on the gel surface. Moreover, the shoulder close to the B-O-C signal at 1125 cm^−1^ would suggest that the boron atom could be involved in this process. In this regard, our result are in agreement with Saeed et al. [40] paper. The authors, using XRD and FTIR techniques, suggest that PVA-borax hydrogel entraps divalent transition metal ions such as Cu^2+^, Fe^2+^ and Zn^2+^ that crosslink complexes of PVA-borax. Specifically, they report that the C-O stretching peak in the presence of Cu (II) and Zn (II) appears at, 1124.50 cm^−1^ and 1118.71 cm^−1^, respectively, and the 1074 cm^−1^ peak was attributed to the B-O-C stretching frequency [40], when divalent transition metal ions are entrapped.

However, it is also important to mention that, in order to deepen this behavior, a higher number of Raman measurements is needed. Furthermore, new experiments with the related statistical analysis are expected to investigate the mechanism of removal of the corrosion products from the different matrices, involving the combination of heteronuclear high resolution NMR and Raman spectroscopy.

### 2.4. SEM-EDS of the Gel Layers Removed after Cleaning of the Stones Surface

The purpose of SEM-EDS analyses was to detect the presence of metals in the hydrogel layer that was peeled off from the soiled stone samples. Metals trapped in the gel network indicate that the PVA-PEO-borax hydrogel was able to remove coin corrosion products from the stone surface.

**Figure 7:**
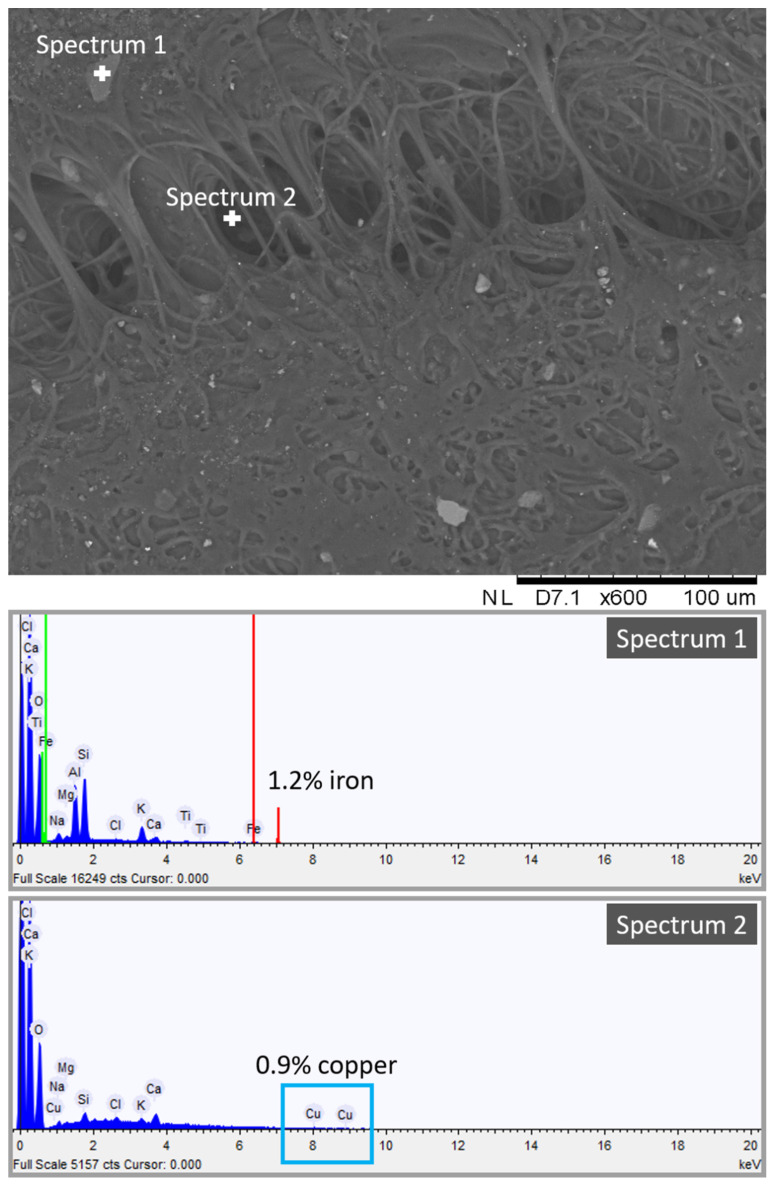
SEM-EDS spectrum and image of the dirty hydrogel layer removed after cleaning of the Travertine surface soiled with acidic corrosion products from penny.

To this end, we obtained a high-resolution image of one dirty gel layer, shown in Figure 7. The heterogeneous porous structure of the PVA-PEO-borax hydrogel is visible in Figure 7. Here, many impurities in the form of crystals trapped into the hydrogel network can be observed. They confirmed the mechanical action of the gel towards particles and dust deposited on the Travertine surface before the soiling process, as suggested by the presence of Ca in the dirty hydrogel layer (see Figure 7), which comes from the Travertine structure made of CaCO_3_. The spectrum of one inclusion revealed the presence of 1.2% of iron (see Figure 7, spectrum 1). Because the composition of the penny coin was copper and zinc, we can suggest that some particles of iron were already present on the stone surface before that the soiling process was obtained by the deposition of acidic corrosion products from the penny. Figure 7 also shows spectrum 2 acquired in a different point, where a 0.9% of copper was detected. The presence of copper is ascribable to the corrosion products of the penny.

SEM-EDS analysis suggested the presence of copper chemically bonded to the hydrogel structure and of iron and other inclusions not chemically bonded to the hydrogel. This indicates that the cleaning procedure of the PVA-PEO-borax hydrogel is both chemical and mechanical. Metal corrosion products are removed by a chemical action, whereas other impurities of the stone surface are removed by a mechanical action. Moreover, the detection of Ca in the hydrogel layer suggests that some particles of CaCO_3_ from the Travertine structure were removed.

### 2.5. Limits of PVA-PEO-Borax Hydrogel to Clean Porous Materials

In this work, the formulation of PVA-PEO-borax in water solution developed by Riedo et al. [20] has been used for the hydrogel preparation. This has shown many weaknesses, in agreement with previous studies [20,21,29,41,42,43]. First of all, it does not seem suitable for the surface cleaning of macro-porous materials, such as the Travertine samples, because of the difficulty in the complete removal of the hydrogel layer, which leads to solid-like residues (see Section 4.1). Despite the fact that the addition of PEO increases the pore size and the liquid phase retention of the gel network, as well as improves its mechanical properties, it has been observed that the residues left on the stone surface may be due to the fact that PEO is the only polymer not chemically bonded to the network [20,21,29,42]. So, PEO seems the major responsible for residues together with the type and state of conservation of the sample analyzed.

NMR, Raman and SEM-EDS results are compatible with a scenario for which metal corrosion products are trapped between two adjacent boron ions as suggested by Saeed et al. [40]. Therefore, the removal efficacy of metals from the sample surface would increase with the increase of boron ions crosslinked to the gel network. This can be achieved thanks to the addition of organic solvents to the PVA-PEO-borax water solution. Indeed, boron ions are insoluble in organic solvents and prefer to be in a cross-linked state [41]. Moreover, it has been noticed that the presence of organic solvents increases the thermal stability of the gel network and the folding of the PVA chains increasing the cross-linking [41].

The PVA-borax hydrogel properties can also be improved by adding another polymer. Some studies [21,43] investigated the effect of agarose on the PVA-borax gel network. Agarose seems to increase the shape stability of the gel. The PVA-borax-agarose gel shows enhanced liquid phase retention and improved mechanical properties [21,43]. The PVA-B/AG gel is more suitable for the cleaning of porous materials, and if it is applied for an adequate contact time, no residues are left [21,43].

## 3. Conclusions

The study we presented here, provides new information for the possible use of PVA-borax hydrogel to clean monuments and marble statues from metal corrosion products. For this reason, we used a portable NMR instrument that allows in situ measurement to study the cleaning efficacy of PVA-borax hydrogel. We confirmed the great potential of single-sided portable NMR as a tool to evaluate the effectiveness of the cleaning procedure. Among the NMR parameters used to investigate the cleaning effectiveness with respect to corrosion products such as copper and iron alloys, as expected, the *T*_1_ relaxation time has proved to be extremely sensitive (it strongly reduces its value in the presence of metallic corrosion products), and therefore we suggest it in a future monitoring protocol with portable NMR instrument.

The NMR, SEM-EDS and Raman data obtained in this work, integrate the data in the literature related to the characterization of PVA-borax hydrogels obtained with multimodal approaches of different microscopic and spectroscopic techniques [20,26,29,40,44]. The NMR study carried out on the surface of Travertine and Carrara marble samples up to 2 mm in deep has shown that is easier to remove PVA-borax hydrogel in Carrara marble compared to Travertine, as Carrara marble is characterized by pores on average smaller with less dispersion than Travertine porosity, which is much more heterogeneous and with the presence of macroscopic pores that prevent the perfect removal of the hydrogel. Therefore, the cleaning action depends on porosity features of stone.

Preliminary experiments of Raman spectroscopy and SEM-EDS performed on PVA-borax hydrogel before and after the cleaning suggest a chemical rather than a physical-mechanical action to remove metal corrosion products. However, for future work, we plan to improve the quality and increase the number of Raman experiments in order to obtain more information.

In conclusion, by combining the NMR, Raman and SEM-EDS results, this work suggests that the mechanism behind the hydrogel cleaning action is to trap metal corrosion products (such as copper or iron, as we used copper coin corrosion for this study) between adjacent boron ions crosslinked with PVA.

## 4. Materials and Methods

Two Travertine samples and one Carrara marble sample with size of 5 × 5 × 2 cm^3^ have been chosen to be studied in this work. Roman Travertine samples have been cut from the same slab and therefore have the same chemical composition characterized by 97–99% CaCO_3_ [45,46]. The Travertines used in this work have been cut parallel to the bedding planes, which means they have larger and more interconnected pores in comparison to samples cut perpendicular to the bedding planes [12,47]. Their porosity is around 6% [48,49]. Intercrystalline and intergranular porosity are poorly sorted and range between 0.01 and 10 μm. Open porosity has a size of up to 100 μm and can achieve a centimetric-size [50]. These macropores are poorly connected [35].

Carrara marble, coming from Carrara region in the central part of Italy, is a calcitic marble mainly composed by calcium carbonate (>99%) but with also low amount of mineral impurities (clay, silt, sand, iron oxide) that produces its characteristic color [31,51,52,53]. Carrara marble is characterized by a total porosity from about 1.6% to 3.3% [31,49,52]. While Travertine has also macropores, Carrara marble shows only micropores and mesopores (0.001 µm < r < 10 µm) [31,50].

Moreover, two different types of coins have been used. One aged penny, which is the British decimal currency composed of copper and zinc, and several 1 euro cent coins made of copper-covered steel.

In order to trigger the acidic corrosion of both penny and euro cent, a solution 0.2 M of citric acid was used. The solution was characterized by pH of 2.5.

**Figure 8:**
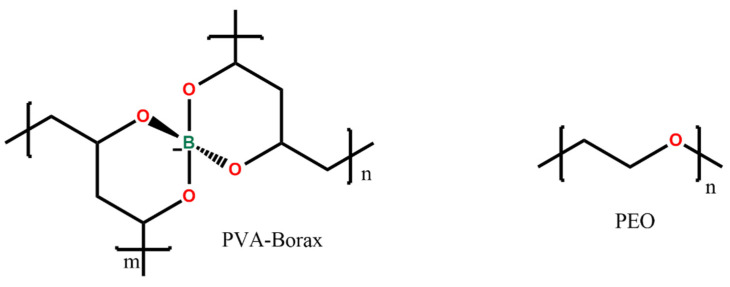
Chemical structure of the hydrogel compounds.

The PVA-PEO-borax hydrogel employed in this research was conceived and described in a previous study by Riedo et al. [20] and investigated by using portable NMR by Stagno et al. [12]. Its components are poly(vinylalcohol) (87–89% hydrolyzed, Mw 85,000–12,400, Sigma-Aldrich, Milano, Italy), poly(ethyleneoxide) (Mw. 37,000–4400, Sigma-Aldrich, Milano, Italy), and sodium tetraborate decahydrate (Sigma-Aldrich, Milano, Italy). In Figure 8, the chemical structure of PVA-borate and of PEO is shown.

### 4.1. Sample Preparation

**Figure 9:**
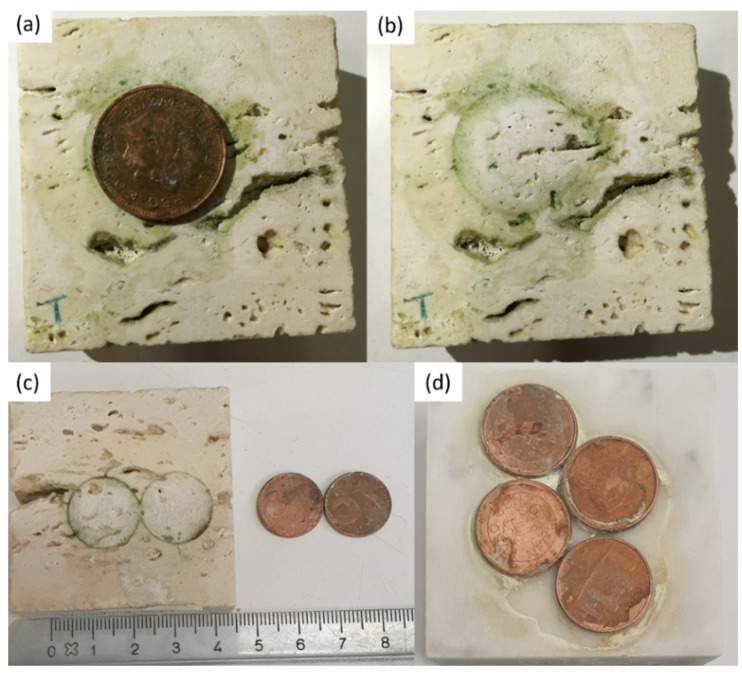
In (**a**,**b**) Travertine sample stained by corrosion products of one penny reacting with citric acid; the greenish-black stain on Travertine produced by reaction of two euro cents with citric acid in (**c**); in (**d**) Carrara marble surface with acidic corrosion of four euro cents with citric acid.

The two Travertine samples have been soiled in different way. On the surface of one Travertine (Travertine 1) an acidic corrosion of one aged penny was induced by using the solution of citric acid (Figure 9a,b), whereas on the surface of the second Travertine sample (Travertine 2) two coins of euro cents with citric acid solution (Figure 9c) have been left to react. The surface of Carrara marble was stained with the corrosion products coming from the reaction of four coins of euro cents with the citric acid solution (Figure 9d).

**Figure 10:**
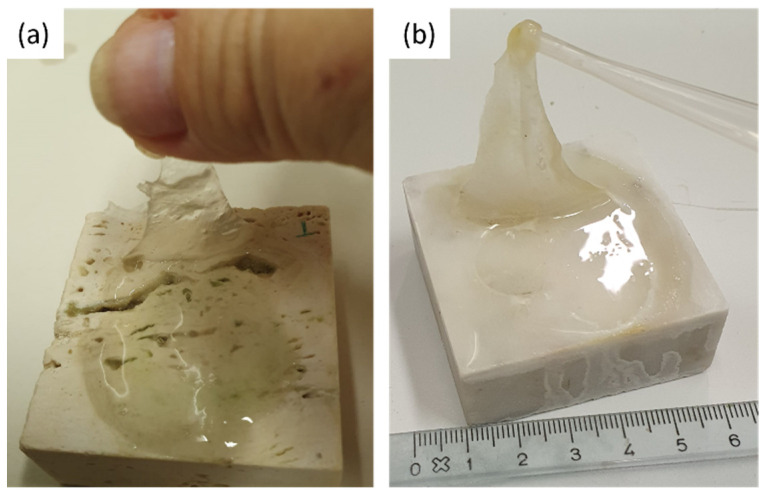
PVA-PEO-borax hydrogel peeling from (**a**) Travertine surface and (**b**) Carrara marble surface.

**Figure 11:**
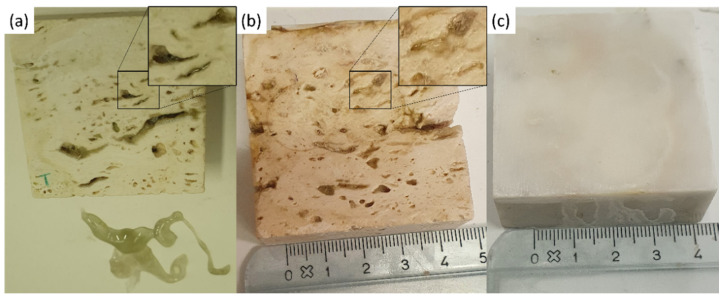
Stone surfaces after PVA-PEO-borax hydrogel cleaning of (**a**) Travertine 1 soiled with corrosion products from penny, (**b**) Travertine 2 soiled with corrosion products from euro cents, and (**c**) Carrara marble soiled with corrosion products from euro cents. In the zoomed portions transparent gel residues are observable in the pores.

The coins and the reagent (i.e., citric acid) were left on the stones surface for one week. After that, the coins were removed from the stones surface and a greenish-black stain was observed (see Figure 9). Each stone surface was treated by the PVA-PEO-borax gel, which was applied in a thin layer and left on the stone surface for 30 min. After this time, the gel layer was peeled off (see Figure 10) and it was left drying and then preserved for Raman analysis. The cleaned stones surfaces (Figure 11) showed a different appearance. The two Travertine samples showed at the naked eye the presence of transparent gel residues in the pores (zoomed portions of Figure 11a,b). All the surfaces were left drying before performing NMR measurements.

### 4.2. Portable NMR Measurements

A Bruker minispec mq-ProFiler with a single-sided magnet that generates a static magnetic field of 0.35 T was used. It was equipped with a RF-probe for performing experiments with a 2 mm depth from the sample surface, characterized by a ^1^H-resonance frequency of 17 MHz and dead time of 2 µs. The two Travertine samples and the Carrara marble sample have been characterized in their natural state by measuring the longitudinal (*T*_1_) and transversal (*T*_2_) relaxation time. *T*_1_ and *T*_2_ of the stones surfaces have been also monitored after the soiling process and after the gel cleaning process. All the NMR measurements have been performed in a controlled environment characterized by a temperature (T) of 25 ± 1 °C and a relative humidity (RH) around 50 ± 3%.

The longitudinal relaxation time (*T*_1_) was acquired by using a Saturation-Recovery (SR) sequence with repetition time (TR) of 0.2 s, 65 steps from 0.05 ms to 8000 ms, increment factor of 1.2 and number of scans (NS) of 2048.

The transversal relaxation time (*T*_2_) was acquired by using a Carr-Purcell-Meiboom-Gill (CPMG) sequence with TR of 1 s, echo time (TE) of 30 µs, 200 echoes, NS = 2048.

Each *T*_1_ and *T*_2_ measurement was repeated three times in order to test the reproducibility and minimize the error.

All data were elaborated by using the Inverse Laplace Transform (ILT) algorithm [54] in MATLAB 2021a to obtain the *T*_1_ and *T*_2_ distribution.

### 4.3. Raman Measurements

The gel layer used to clean each surface of the stones from the corrosion products was analyzed by using Raman spectroscopy. The instrumental setup is represented by a Horiba Jobin-Yvon HR Evolution micro-Raman spectrometer. This is equipped with a He-Ne laser (λ = 632 nm), coupled with a microscope with a set of interchangeable objectives. A 100× objective was used. Intensity of radiation has been set at 15 mW by neutral filters; the acquisition time has been varied between 10 and 15 s for each scan, and the number of acquired scans has been changed between 30 and 60 scans. Images of the pure gel and of the gel layers removed from Travertine and Carrara marble have been acquired with 100× magnification. Moreover, spectra were acquired in three or five different points of pure gel and of gel used to clean Travertine soiled with corrosion products from penny and Carrara marble soiled with corrosion products from euro cents.

### 4.4. SEM-EDS Measurements

The gel layer removed from the Travertine stone after cleaning of the penny corrosion products was analyzed by using a TM 3000 HITACHI scanning electron microscope (SEM) equipped with an EDX SWIFT ED 3000 probe. No coating treatment was applied to the sample. The accelerating voltage was 15 kV. From the sample, an image with a 600× magnification and an acquisition time of 10 min was acquired. Then, several points on the image were detected and the elemental composition in each point was obtained. The acquisition time of each spectrum was 3 min.

## Figures and Tables

**Table 1:** Tentative assignations of observed Raman peaks for PVA-borax hydrogel.

Wavenumber	Assignation
1125	B-O-C stretching
1355	C-H bending
1440	C-H bending
2855	C-H stretching
2913	C-H stretching
2935	C-H stretching

## Data Availability

The data presented in this study are available on request from the corresponding author.
